# Efficient simultaneous removal of heavy metals and polychlorobiphenyls from a polluted industrial site by washing the soil with natural humic surfactants

**DOI:** 10.1007/s11356-021-12484-x

**Published:** 2021-01-20

**Authors:** Alessandro Piccolo, Antonio De Martino, Francesco Scognamiglio, Roberto Ricci, Riccardo Spaccini

**Affiliations:** 1 Centro Interdipartimentale di Ricerca sulla Risonanza Magnetica Nucleare per l’Ambiente, l’Agro-Alimentare ed i Nuovi Materiali (CERMANU), Via Universita 100, 80055 Portici, NA Italy; 2 Dipartimento di Agraria, Università di Napoli Federico II, Via Universita 100, 80055 Portici, NA Italy; 3 Biosearch Ambiente srl, Via Tetti Gai, 59, 10091 Alpignano, TO Italy

**Keywords:** Industrial site, Soil washing, Humic acids, Heavy metals, Polychlorobiphenyls

## Abstract

We evaluated the effectiveness of natural organic surfactants such as humic acids (HA) from lignite to simultaneously wash heavy metals (HM) and polychlorobiphenyls (PCB) from a heavily contaminated industrial soil of northern Italy. Supramolecular HA promote in solution a micelle-like structure, where recalcitrant apolar organic xenobiotics are repartitioned from surfaces of soil particles during soil washing process. Concomitantly, the HA acidic functional groups enable a simultaneous complexation of HM. A single soil washing with HA removed 68 and 75% of PCB congeners for 1:1 and 10:1 solution/soil ratios, respectively. The same HA washing simultaneously and efficiently removed a cumulative average of 47% of total HM, with a maximum of 57 and 67% for Hg and Cu, respectively. We showed that washing a highly polluted soil with HA solution not only is an effective and rapid soil remediation technique but also simultaneously removes both HM and persistent organic pollutants (POP). Soil washing by humic biosurfactants is also a sustainable and eco-friendly technology, since, contrary to synthetic surfactants and solvents used in conventional washing techniques, it preserves soil biodiversity, promotes natural attenuation of unextracted POP, and accelerates further soil reclamation techniques such as bio- or phytoremediation.

## Introduction

Malfunctioning of industrial activities often causes contamination of soils and waters by toxic and persistent organic pollutants (POP), such as polychlorobiphenyls (PCB) and/or heavy metals (HM). PCB are among the most hazardous pollutants (Borja et al. 2005), and, as highly hydrophobic contaminants, are adsorbed in soils on surfaces of clay and other oxides and on organic matter (Wang et al. 2008), thereby becoming hardly bio-accessible and, hence, very poorly biodegradable (Field and Sierra-Alvarez 2008). HM are inorganic pollutants whose excessive accumulation in soil may induce phytotoxicity, limit microbial activity, and affect soil functions (Dussault et al. 2008). Moreover, leaching of HM from soil can contaminate waters, bioaccumulate in aquatic organisms, and eventually enter the animal and human food chain (Shaker and Albishri 2014). Pollution by PCB and HM is prevalently found around industrial sites where their concentration can be several orders of magnitude larger than allowed by the laws of different countries (Mackova et al. 2006). In Italy, for example, all soils containing more than 5.0 mg kg^−1^ of PCB (D. Lgs 152/2006) must be decontaminated.

Since most common practices of elimination of recalcitrant pollutants, such as soil incineration or burial in disposal sites, are very expensive and involve losses of fertile soils (Trellu et al. 2016), alternative soil remediation methods are required to be concomitantly efficient but less expensive and disruptive of soil qualities (Focht and Reineke 2002). In recent years, ex situ soil washing (SW) or in situ soil flushing (SF) technologies, based on the desorption of pollutants by inorganic and organic acids, chelating agents, and natural or synthetic surfactants, often coupled to selected microbial cultures, have been applied as remediation technologies because of rapid cleanup and cost reduction (Lászlová et al. 2018).

However, the outcome of a remediation process depends on the physical–chemical and biological characteristics of soil (Jensen et al. 2011; Tsang and Hartley 2014), but also on the binding strength of pollutants on the organic and inorganic soil components. To increase their solubility and improve soil decontamination, strong complexing agents or acid and alkaline solutions are often employed in soil washing processes (Jean et al. 2007; Zhang et al. 2007). However, extreme pHs and synthetic surfactants can alter soil characteristics and functions and become toxic to soil biota (Bianchi et al. 2008; Schmidt and Brauch 2004). For example, nitrilotriacetic acid and citrate, though effective soil washing agents for As and Cu removal can be phytotoxic and degrade water quality when reaching ground and surface waters by leaching and runoff (Rasmussen et al. 2015).

Biosurfactants are considered more sustainable and ecologically compatible than synthetic surfactants for washing soils contaminated by organic pollutants (Ying 2006; Trellu et al. 2016). Their removing capacities are due to the formation of micellar structure, whereby the hydrophobic core pulls the organic contaminants off their adsorption sites (Chandler 2005). Natural biosurfactant are humic acids (HA), the most stable fraction of soil humus and of geochemical organic deposits. HA are a complex supramolecular associations of relatively small and heterogeneous molecules, which have survived the biotic and abiotic degradation of biomolecules released from lysed cells (Piccolo et al. 2018), and their superstructural conformation is stabilized by hydrogen bonds, hydrophobic interactions, and electrostatic bonds bridged by metals (Piccolo 2016; Lipczynska-Kochany 2018; Wells 2019). The presence of both hydrophobic and hydrophilic systems in HA (Nebbioso et al. 2014) enables the formation of pseudo-micellar structures whose critical micelle concentrations (CMC) are reported to vary from 5 to 10 g L^−1^ (Guetzloff and Rice 1994; Tombácz 1999; Smejkalova and Piccolo 2008**)**.

The biosurfactant properties of HA from lignite were used to wash two different soils from an industrial site heavily contaminated with organic pollutants (Conte et al. 2005). The removal of highly toxic pollutants (PAHs, monoaromatic halogenated and nitrogenated compounds, thiophenes, sulphones, biphenyls) reached more than 80% and performed equally, if not better, as that of two synthetic surfactants, such as SDS and Triton X-100. Fava and Piccolo (2002) showed that the aerobic biodegradation and dechlorination of PCB in a model soil inoculated with bacteria were made possible only by the presence of HA, which enabled a 150% more efficient PCB disappearance than in soil without HA. Similarly, HA were found to significantly improve the aerobic remediation of a soil contaminated by PAH (Fava et al. 2004).

The remediation of HM contaminated soils was tried by a number of biodegradable chelators (Wang et al. 2020). The significant complexing capacity of HA from different sources make these natural materials particularly useful for washing soils polluted with HM (Tsang and Hartley 2014; Mao et al. 2015; Meng et al. 2017). However, the efficiency of washings was shown to depend on the molecular composition of HA and its complexing properties (Piccolo et al. 2019a).

The aim of this work was to evaluate for the first time the capacity of an aqueous solution of a humic surfactant isolated from Leonardite to simultaneously remove both HM and PCB from a highly polluted soil of an industrial site of Northern Italy by the soil washing technique, and to test the efficiency of contaminants removal at two different soil solution ratios.

## Materials and methods

### Humic acid

A HA was isolated from a lignite sample of North Dakota Leonardite (Mammoth, Chem. Co., Houston, Texas), and purified as reported elsewhere (Piccolo 1988). The HA was then suspended in distilled water and titrated to pH 9 by an automatic titrator (VIT 909 Videotitrator, Copenhagen) with a 0.1 M NaOH solution under N_2_ stream. The resulting sodium-humate was then filtered through a Millipore 0.45 μm, freeze-dried, and analyzed for elemental content by a Fisons EA 1108 Elemental Analyzer. This HA contained 2.7% of ashes, 56% C, 4% H, 2% N, and, by difference, 38% O or other elements. The carbon distribution and both hydrophobicity and aromaticity of this Leonardite HA, as obtained by ^13^C-CPMAS-NMR spectroscopy, are shown elsewhere (Spaccini et al. 2002).

### Soil and soil washing experiments

The soil was collected (30 cm depth) by Biosearch Ambiente srl from the highly polluted Brescia-Caffaro industrial site located in Northern Italy (http://bresciacaffaro.it/sito-bonifica-brescia-caffaro.html). The Caffaro plant produced PCB and PCB mixtures (Fenclor, Fenclor DK, and Apirolio) until 1984 and the surrounding areas were found to be heavily contaminated by PCB but also PCDDs, PCDFs, DDT and its isomers, metalloids and metals (e.g., As and Hg) (Di Guardo et al. 2017). After extensive homogenization, the soil sample was air-dried and sieved through a 2.00-mm sieve and characterized for basic physical–chemical properties by common methods, providing the following characteristics: pH (CaCl_2_) 7.5; sand 43.4%; silt 41.4 %; clay 15.2%; organic carbon (OC) 0.61%; total nitrogen (N) 0.12%.

Soil washing (SW) experiments for the removal of PCB were conducted in glass bottles by adding either 10 L (liquid to solid ratio, L/S, of 10:1 L kg^−1^) or 1 L (L/S of 1:1 L kg^−1^) of a 10 g L^−1^ solution of HA to 1 kg of contaminated soil. A control experiment was conducted by washing the soil with only MilliQ grade water at the same L/S ratios used for the contaminants removal. The glass bottles were placed in a horizontal shaker and shaken at 150 rpm for 24 h. The suspension was then centrifuged at 3000 rpm for 30 min, the soil residue was re-suspended with MilliQ grade water, centrifuged again, and the supernatants containing the HA solutions discarded. The soil residue was air-dried and finely powdered for the determination of PCB and HM content. Each experiment was performed in triplicate.

### Determination of PCB

PCB were determined by a modified EPA Method 1668C (2010). Briefly, 25 g of soil before and after washing by either water or HA solutions were spiked with 50 μL of 2,4,5,6-tetrachloro-m-xylene solution at a concentration of 10 mg L^−1^, added with 50 ml of *n*-hexane and submitted to PCB extraction in a ultrasonication bath (SONICA, 3200M S3, of Soltec) for 30 min. After sonication, the suspension was cooled at room temperature and the organic phase filtered through Whatman 42 filter paper containing sodium sulfate at the top of the filter in order to remove residual water. The extraction was repeated twice, and the organic extracts were combined, and rotoevaporated to a final volume of 10 mL. A further purification was achieved by loading 2 mL of the organic extract through a Bond Elut Florisil cartridge (1 g/6 mL, Agilent Technologies), previously conditioned with 10 ml of *n*-hexane, and, then, eluted with 10 mL of fresh *n*-hexane. This volume was further concentrated to 2 mL and added, before GC-MS analysis, with 10 μL of a quintozene (Supelco) 1 mg mL^−1^ solution in hexane as internal standard.

The determination of PCB was accomplished by a GC-MS system consisting of an Agilent 7890 gas-chromatograph, equipped with a split/splitless injector, a HP-5MS capillary column (30 m × 0.25 mm, Agilent Tech., USA), and an Agilent 5975B mass spectrometry detector. The experimental conditions for GC analyses were the following: (1) initial temperature of 80 °C, hold time 0 min.; (2) rate of 3 °C min^−1^ up to 250 °C, hold time 0 min; (3) rate of 3 °C min^−1^ up to 300 °C, hold time 2 min. The total GC run time was 63.66 min. Helium was the carrier gas at 1.0 mL min^−1^ and the splitless-flow was used. The inlet-line temperature of the GC was set at 250 °C, MS source at 150 °C and the mass transfer line at 300 °C. A solvent delay time of 5 min was applied before spectra acquisition to reduce filament consumption. Mass spectrometer operated in electron impact ionization (EI)/selected ion monitoring (SIM) mode. Three PCB standard solutions (dr. Ehrenstorfer GmBh) were used for qualitative and quantitative analysis, at 10 ng μl^−1^ in iso-octane containing the following PCB congeners: 1. 77, 81, 105, 114, 118, 123, 126, 156, 157, 167, 169, and 189; 2. 28, 52, 95, 99, 101, 105, 110, 118, 138, 146, 149, 151, 153, 170, 177, 180, 183, and 187; 3. PCB 209. The 2,4,5,6-tetrachloro-m-xylene (CPAchem Ltd) at 100 ng μl^−1^ in methanol and quintozene were used as spike and internal standard, respectively. Qualitative analysis was performed by comparing retention time and m/z of PCB congeners occurring in the certified standard mixtures with GC-MS peaks, while quantitative analysis was achieved by using the GC response factor of each target PCB obtained with five-point calibration curves of the same certified mixture standard. Each sample was injected twice and average and standard deviation were calculated.

### Determination of heavy metals

Soil samples before and after soil washings were ground using a PM 20 ball mill (Retsch) and then mineralized in a microwave (Milestone, Digestor/ Dring Ethos 900). All glassware and plastic ware used in the mineralization were previously acid-washed with 3 M HCl and rinsed with MilliQ grade water. A sample of about 0.5 g was accurately weighed into a PTFE pressure vessel together with 9 mL of HCl (37%) and 3 mL of HNO_3_ (65 %). After microwave digestion, the solutions were cooled, filtered through Whatman 42 filter paper and diluted with MilliQ grade water up to 25 mL. The amount of HM was determined by using of an atomic absorption spectrometry (AAS, Perkin–Elmer model 3030 B) connected to a FIAS 100 flow injection system. Most HM were determined by conventional flame AAS, while arsenic and mercury were measured, respectively, through a flow injection hydride generation using NaBH_4_ to generate arsenic hydride and a cold vapor flow injection using NaBH_4_ as reductant agent. Stock standard solutions of HM are obtained from BDH Reagents (Poole, UK). Standard procedures as recommended by the manufacturers were utilized. The average of three absorption measurements was recorded for each sample.

## Results and discussion

### Characteristics of humic acid and soil

The HA from a Leonardite used in this study was already proved to be an efficient biosurfactant for the washing of organic pollutants from soils (Conte et al. 2005). Its capacity to remove organic contaminants from soils is due to its large content of methylenic chains and aromatic components, as evaluated by ^13^C-CPMAS-NMR spectroscopy, that confer to this HA a great degree of hydrophobicity (Spaccini et al. 2002) and the capacity to form pseudo-micellar domains (Smejkalova and Piccolo 2008; Chilom et al. 2009). The same weak forces, including hydrogen bonding, van der Waals interactions, charge transfer and hydrophobic *π*–*π* bonds that stabilize the humic supramolecular conformations (Piccolo 2002), facilitate the incorporation of organic xenobiotics into humic pseudo-micelles during the soil washing process (Lipczynska-Kochany 2018; Piccolo et al. 2019b). The chemical affinity with humic molecules and the poor water solubility of recalcitrant organic pollutants foster their selective repartition from the soil solid surfaces into the pseudo-micellar hydrophobic domains of HA.

On the other hand, the considerable content of dissociated carboxyl and phenolic functional groups of this HA (Spaccini et al. 2002) at a pH of 9, is responsible for the chelation of metal ions and their efficient removal from soil in the washing process (Piccolo et al. 2019a). Therefore, the peculiar characteristics of HA, that concomitantly exert both micellar and chelating properties, enable the simultaneous soil reclamation of both organic and metal contaminants by washing with a single biosurfactant.

### Soil washing of heavy metals

Preliminary studies on the best liquid to soil ratios have shown that the 10:1 ratio provided the most efficient removal of HM by soil washings with humic biosurfactant solutions from a clayey soil spiked with different concentrations of HM (Piccolo et al. 2019a). The same liquid to soil ratio was applied here to remove HM by a humic solution from the sandy-loamy soil at the industrial site of the ex-Caffaro plant near Brescia, Italy. The amount of HM remaining in soil after washing with either HA solution or water alone is shown in Table 1. The HA solution removed from soil a significant larger amount of HM that went from 29% for As up to 57 and 67% for Hg and Cu respectively, but showing a cumulative average removal of 47% for all metals. Moreover, the increase of removal by the humic biosurfactant in respect to water alone went from 7% for As to as high as 43% for Hg, and was up to an average of 20% for the 8 heavy metals searched here (Table 1).

**Table 1:** Amount (mg kg^−1^) and percentage (%) of heavy metals (HM) in soil before and after washing by either HA solution or water at liquid:solid ratio of 10:1

HM	HM in original soil	Amount of HM in soil after washing		Percentage of HM removed with washing	
		HA	H_2_O	HA	H_2_O
As	55 ± 7^a^	39 ± 4^b^	43 ± 4^ab^	29	22
Co	17 ± 1^a^	12 ± 1^b^	16 ± 1^ac^	29	6
Cr	28 ± 4^a^	17 ± 4^b^	21 ± 4^ab^	39	25
Cu	99 ± 9^a^	33 ± 4^b^	55 ± 6^c^	67	44
Hg	7 ± 1^a^	3 ± 0.2^b^	6 ± 1^ac^	57	14
Ni	41 ± 5^a^	24 ± 4^b^	30 ± 3^bc^	41	27
Pb	214 ± 10^a^	85 ± 11^b^	122 ± 10^c^	60	43
Zn	221 ± 11^a^	100 ± 11^b^	139 ± 16^c^	55	37

Row values followed by a different letter indicate significantly different values (*P* < 0.05), as determined by one-way ANOVA followed by the Tukey test

HM in soils can be readily available in the soil solution, exchangeable on the soil exchange sites, complexed by the organic matter, and occluded into soil oxides or in the lattice structure of primary and secondary soil minerals (Rao et al. 2007). Therefore, the strong bindings of HM to soil components or their oxide forms reduce their bioavailability and affect the efficiency of washing process. Nevertheless, humic biosurfactants are reported to efficiently mobilize labile, exchangeable, and complexed HM due to their large complexing capacities (Halim et al. 2003; Garcia-Mina 2006). The supramolecular conformation of HA coupled to their negatively charged acidic functional groups were shown to improve the mobilization of As, as arsenate [As(V)], and other metals such as Zn, Pb, and Cu from a mine tailings by soil flushing with HA, which also reduce metal precipitation (Wang and Mulligan 2009). A humic acid from sewage sludge compost at a concentration of 3000 mg of C L^−1^ was used to wash a sandy soil artificially spiked with 1984 mg kg^−1^ of Cu and 50 mg kg^−1^ of Cd and found to remove by a single washing 80.7% of Cu and 69.1% of Cd (Kulikowska et al. 2015). Furthermore, it has been shown that ten repeated washings of a moderately contaminated loamy soil by HA at a concentration of 100 mM C, and a solution:soil ratio of 10:1 removed 16% of As and 61% of Cu (Rasmussen et al. 2015). However, a comparison of the metal washing capacity of humic matter isolated from Leonardite with that extracted from different composts indicated that the amount of metals removed by soil washing was a function of the molecular characteristics of humic biosurfactants and, in particular, of their content of carboxyl functions (Piccolo et al. 2019a). In fact, in the same study, it was shown that a compost made with a larger proportion of straw had similar, if not better, metals washing performances as the HA from Leonardite, thereby indicating that this washing technique for soil HM removal is also sustainable due to the low cost and large abundance of green compost, from which the required humic biosurfactants are obtained.

As in previous studies (Halim et al. 2003; Warwick et al. 2005; Piccolo et al. 2019a), the percent of HM removal obtained here by soil washing with either the humic solution or water alone generally followed the Irving and Williams (1953) series of stability constants of divalent metals, with Pb, and Cu being displaced by a greater extent than Zn (Table 1). Our results further indicate the large capacity of the HA solution to wash away Hg from soil (65%). This increased Hg solubilization is attributed to the formation of multi-dentate complexes between HA and Hg (Garau et al. 2007; Padmavathiamma and Li 2010). In fact, based on the its complexation capacity, humic matter has been shown to significantly reduce the emission of Hg from a sandy soil (Mauclair et al. 2008) and increase plant tolerance to Hg contamination of soils (Cozzolino et al. 2016).

### Soil washing of polychlorobiphenyl

**Fig. 1:**
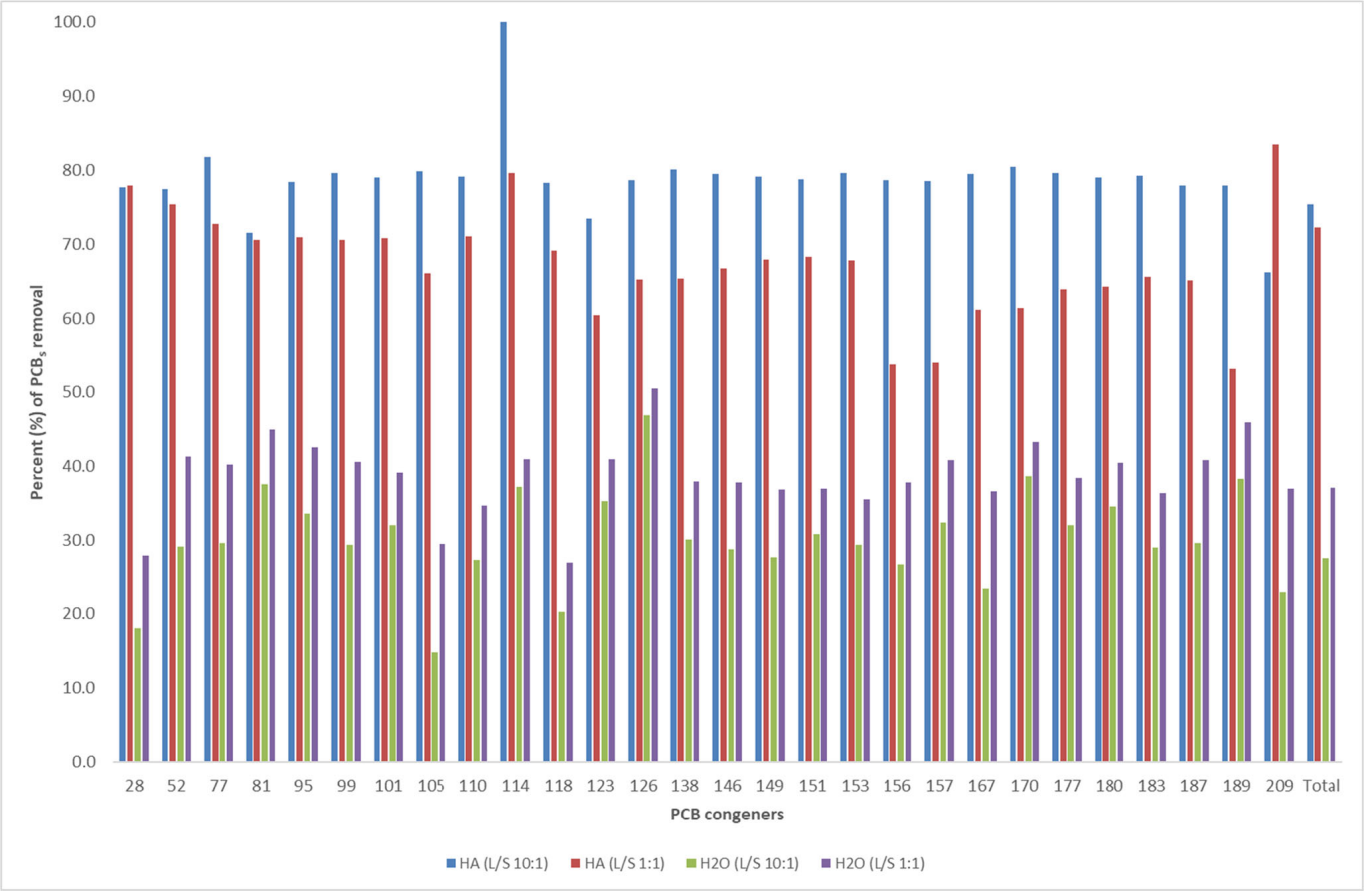
Percentage (%) of single PCB removal after soil washing with humic acid and water alone at ratio liquid:solid (L/S) of 10:1 and 1:1

The overall concentration of PCB in this polluted soil amounted to 5345 μg kg^−1^ and the most abundant congener was the deca-CB with 1569 μg kg^−1^ (Table 2). Washing only by water left an amount of PCB in soil that was 3362 and 3871 μg kg^-1^ for the liquid to soil (L/S) ratio of 10:1 and 1:1 (Table 2), corresponding to a total removal percentage of 37% and 28% (Fig. 1), respectively. While these results show a tendency of the 10:1 L/S to wash off more PCB than the 1:1 ratio, the two ratios were not significantly different for all congeners, except for the tetra-CB 77 and deca-CB 209 (Table 2).

**Table 2:** Amount of PCB (μg kg^−1^) in soil before and after washing by either HA solution or water at liquid:solid ratio of either 10:1 or 1:1

PCB congeners	PCB in original soil	PCB in soil after washing with HA solution		PCB in soil after washing with water	
		Ratio 10:1	Ratio 1:1	Ratio 10:1	Ratio 1:1
Tri-CB					
28	163 ± 13^a^	37 ± 5^b^	36 ± 11^b^	118 ± 4^c^	133 ± 12^c^
Tetra-CB					
52	142 ± 5^a^	32 ± 2^b^	35 ± 9^b^	83 ± 6^c^	100 ± 9^c^
77	6 ± 0.1^a^	1 ± 0.2^b^	2 ± 0.2^c^	4 ± 0.1^d^	4 ± 0.3^e^
81	3 ± 0.1^a^	1 ± 0.1^b^	1 ± 0.1^b^	2 ± 0.1^c^	2 ± 0.2^c^
Sum	151	34	38	89	106
Penta-CB					
95	143 ± 3^a^	31 ± 1^b^	42 ± 10^b^	82 ± 8 ^c^	95 ± 9^c^
99	88 ± 3^a^	18 ± 1^b^	26 ± 6^b^	52 ± 3^c^	62 ± 5^c^
101	255 ± 8^a^	54 ± 1^b^	74 ± 17^b^	155 ± 23^c^	173 ± 12^c^
105	76 ± 5^a^	16 ± 1^c^	26 ± 8^c^	54 ± 8^b^	65 ± 11^ab^
110	216 ± 6^a^	45 ± 2^b^	63 ± 15^b^	141 ± 18^c^	157 ± 20^c^
114	4 ± 0.5^a^	0^b^	1 ± 0.1^b^	2 ± 0.3 ^a^	3 ± 0.2^a^
118	213 ± 9^a^	46 ± 1^b^	6 ± 17^b^	155 ± 8^c^	170 ± 9^c^
123	11 ± 2^a^	3 ± 0.2^b^	4 ± 0.8^bc^	6 ± 0.5^c^	7 ± 0.6^c^
Sum	1006	213	242	647	732
Hexa-CB					
126	11 ± 0.5^a^	2 ± 0.1^b^	4 ± 0.6^b^	5 ± 0.2^c^	6 ± 2^c^
138	482 ± 24^a^	96 ± 5^b^	167 ± 40^c^	299 ± 14^d^	337 ± 31^d^
146	44 ± 1^a^	9 ± 0.5^b^	15 ± 3^b^	27 ± 5^c^	31 ± 5^c^
149	278 ± 10^a^	58 ± 3^b^	90 ± 20^c^	176 ± 17^d^	201 ± 7^d^
151	114 ± 3^a^	24 ± 1^b^	36 ± 8^b^	72 ± 6^c^	79 ± 11^c^
153	443 ± 24^a^	91 ± 7^b^	143 ± 32^b^	286 ± 8^c^	313 ± 31.^c^
156	35 ± 3^a^	7 ± 0.1^b^	16 ± 6^bc^	22 ± 1^cd^	25 ± 7^acd^
157	11 ± 0.8^a^	2 ± 0.2^b^	5 ± 2^bc^	7 ± 0.5^cd^	8 ± 0.6^cde^
167	14 ± 0.4^a^	3 ± 0.5^b^	5 ± 2^c^	9 ± 0.5^d^	11 ± 2^d^
Sum	1432	292	481	903	1011
Hepta-CB					
170	190 ± 9^a^	37 ± 4^b^	73 ± 17^c^	108 ± 4^d^	116 ± 18^d^
177	80 ± 4^a^	16 ± 1^b^	29 ± 6^c^	49 ± 3^d^	55 ± 6^d^
180	458 ± 16^a^	96 ± 7^b^	164 ± 35^c^	273 ± 26^d^	300 ± 6^d^
183	230 ± 8^a^	48 ± 4^b^	80 ± 17^c^	147 ± 11^d^	164 ± 12^d^
187	59 ± 2^a^	13 ± 1^b^	21 ± 5^c^	35 ± 2^d^	42 ± 6^d^
189	7 ± 0.6^a^	2 ± 0.2^b^	3 ± 0.7^c^	4 ± 0.4^cd^	4 ± 0.3^cd^
Sum	1024	212	370	616	681
Deca-CB					
209	1569 ± 21^a^	530 ± 15^b^	515 ± 46^b^	989 ± 44^c^	1208 ± 25^d^
Total	5345	1318	1682	3362	3871

Row values followed by a different letter indicate significantly different values (*P* < 0.05), as determined by one-way ANOVA followed by the Tukey test

Except for less chlorinated congeners (Cl < 5), which are sparingly soluble, it is known that PCB are scarcely soluble in aqueous solution, and they can be present in soil in three fractions: readily available, potentially available, and bound to the soil matrix (Vasilyeva and Strijakova 2007). The latter fraction is prevalently adsorbed on the surface of fine colloidal soil particles, due to the greater surface to volume ratio and organic matter content than for coarser particles. The unusual relatively large PCB removal in water may be explained by the application of ultrasonic vibrations that increased the amount of PCB-rich soil particles suspended in water. In fact, it has been shown that ultrasonic vibrations increase the efficiency of pollutants removal from soil in respect to soxhlet extraction because the ultrasonic disruption of macroaggregates exposes the inner fine particles to an enhanced pollutants solubilization (Conte et al. 2005). Moreover, the larger aqueous solution used in the extraction by the 10:1 L/S ratio favored the partition in water of PCB present in the readily and potentially available fractions.

**Fig. 2:**
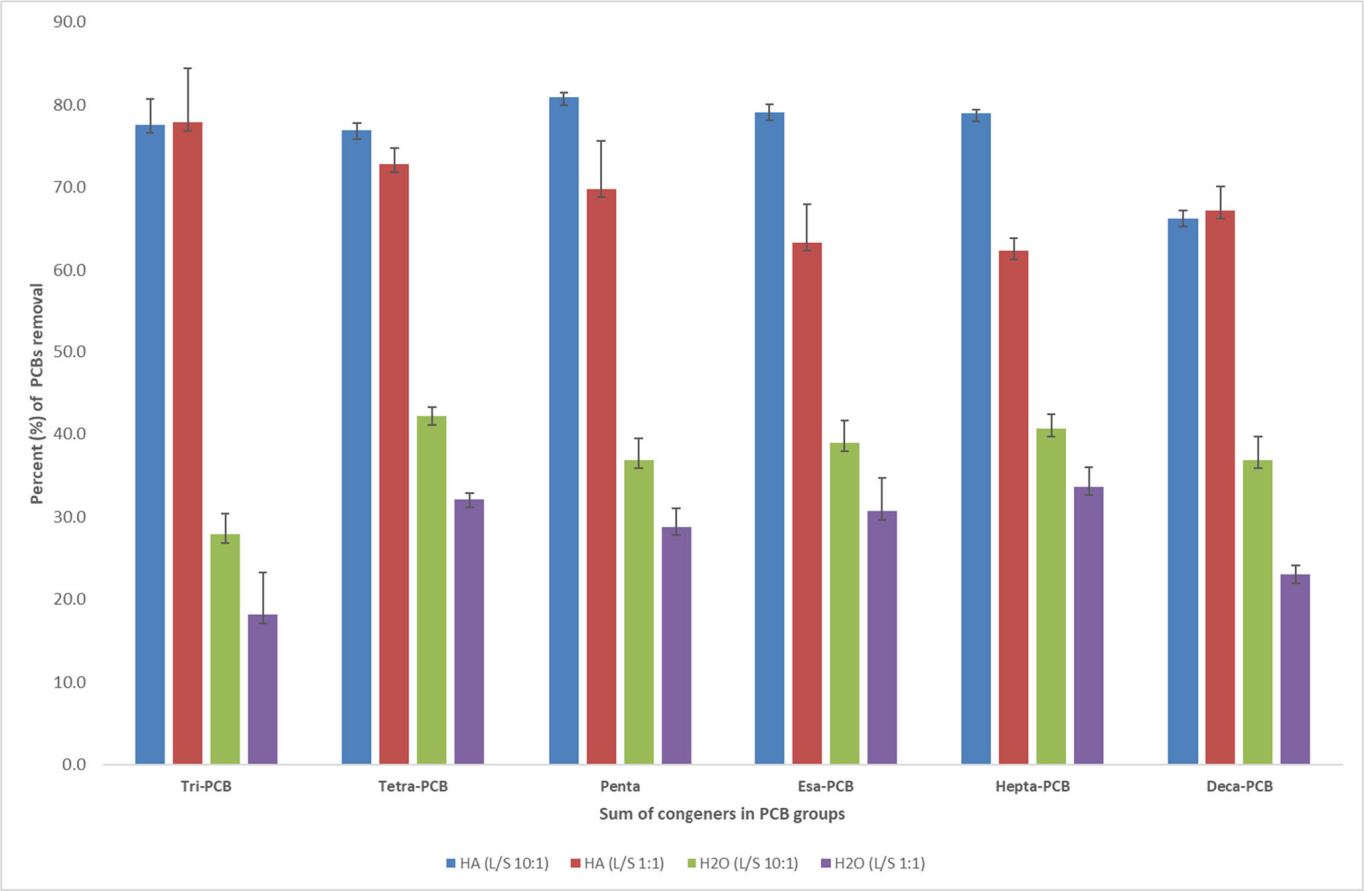
Average percentage (%) of PCB removed in different groups after soil washing with humic acid and water alone at ratio liquid:solid (L/S) of 10:1 and 1:1

When the washing was conducted with the humic solution, the amount of total PCB removed from soil was much greater than by washing with water alone (Table 2). In fact, the content of total PCB remaining in soil after the HA washing was 1318 μg kg^−1^ for the L/S ratio of 10:1, and 1682 μg kg^−1^ for 1:1 (Table 2), corresponding to a total removal percentage of 75% and 69%, respectively (Fig. 1). In particular, the soil washing by HA solution showed a similar removal efficiency with both 10:1 and 1:1 L/S ratios for the tris- (77 versus 78%) and deca-CB (66 versus 67%), whereas it was greater with the former than for the latter ratio for tetra- (78 versus 74%), penta- (79 versus 76%), hexa- (80 versus 66%), and hepta-CB (79 versus 64%) (Fig. 2).

The capacity of surfactants to enhance the mobilization of organic pollutants from soil has been already indicated and attributed to an increased pseudo-solubility from the solid phase into the micellar phase of surfactants (Chandler 2005; Pacwa-Płociniczak et al. 2011). HA remove organic contaminants from polluted soils by favoring the repartition of pollutants into the hydrophobic pseudo-micellar domains formed in water by humic matter (Conte et al. 2005; Lipczynska-Kochany 2018). The formation of micellar structures in humic solutions was shown by Dosy-NMR spectroscopy (Smejkalova and Piccolo 2008), while Smejkalova et al. (2009) reported that HA with a high aromatic character reduced the NMR molecular mobility of 2,4-dichlorophenol and 2,4,6-trichlorophenol as a consequence of their inclusion and trapping within HA hydrophobic cavities. The efficient mechanism of repartition of several recalcitrant organic pollutants from the soil solid phase into the humic pseudo-micellar domains of a humic solution was earlier shown when the contamination of a heavily polluted soil was reduced with a single HA washing by about 80%, an efficiency that was similar if not better than by soil washing with synthetic surfactants (Conte et al. 2005; Sannino et al. 2013).

The soil washing of PCB by HA solutions may result not only more efficient but also much faster than the bioremediation processes. In fact, although microorganism by anaerobic and aerobic treatments have been used to promote PCB biodegradation in soil, this process is not only time consuming but also poorly efficient as it depends on several chemical and environmental factors (Furukawa and Fujihara 2008) such as nutrients availability, temperature, pH (Borja et al. 2005; Wiegel and Wu 2000), and type of bacteria (Sannino et al. 2016). Moreover, aerobic microorganisms degrade prevalently lower chlorinated PCB congeners while anaerobic microbes are known to transform highly and ortho-chlorinated PCB (Adebusoye et al. 2008; De et al. 2006). In addition, in many cases PCB dechlorination occurs only after a lag period that varies from few days to several months and is also liable to generate in the process further toxic by-products (Wiegel and Wu 2000; Chen et al. 2001).

Similarly, the soil washings with humic surfactants appear much more efficient than the phytoremediation progressively called upon to reclaim polluted soils, since not only the latter method explores a very thin soil depth but also its contribution to the microbial degradation of PCB is extremely slow (Van Aken et al. 2010; Jing et al. 2018). In fact, it has been estimated that the phytoremediation of an industrial soil contaminated by relatively biolabile PCB, such as tri- and tetra-CB congeners, would take more than 20 years to be completed (Schwitzguébel 2017). Conversely, this work has shown that a single and rapid washing with HA solutions at a L/S ratio of 10:1 was equally effective in removing either low- or high-chlorinated PCB congeners and either planar or no planar PCB. In fact, our findings do not reveal any relation between number and position of chlorine atoms and amount of PCB removed from soil, thereby indicating that the soil decontamination by HA washing is substantially independent on type of PCB congeners.

## Conclusions

Soil washing by solutions of humic surfactants can be a promising technology for rapid, efficient, and simultaneous remediation of soils contaminated by both heavy metals and very recalcitrant pollutants, such as PCB. In fact, the HA washing removed from soil an average of 47% of all HM, with a peak of 57 and 67% for highly toxic metals such as Hg and Cu. The much larger efficiency of HA than water alone in removing HM from soil is due to the considerable chelating capacity of the heterogeneous acidic functional groups present in the humic molecules.

Concomitantly, the HA surfactant property leads to formation of pseudo-micellar domains when the supramolecular structure of humic matter is arranged in aqueous solutions, and favors the repartition of highly hydrophobic pollutants like PCB from the soil surfaces into the humic pseudo-micelles. In fact, the same soil washing experiment that efficiently removed HM, also enabled the removal of up to 75% of the total PCB present in the industrially polluted soil that was investigated here.

We thus believe that the use of natural humic surfactants represents an efficient, environmentally friendly, and cost-effective alternative to common methods for soil remediation and may well replace commercial synthetic surfactants in soil washing technologies. In fact, a washing procedure based on humic solutions can be applied to remove simultaneously from soils large amounts of both heavy metals and recalcitrant organic compounds and the resulting contaminated HA easily disposed by an ex situ incineration. Moreover, the washing by humic surfactants would leave behind in soil enough metabolic carbon to speed up the natural attenuation of the organic pollutants remaining in soil.
